# Point-of-care Ultrasound for the Evaluation of Acute Arterial Pathology in the Emergency Department: A Case Series

**DOI:** 10.5811/cpcem.2021.11.54904

**Published:** 2022-01-28

**Authors:** Aaran Drake, Nicholas Dreyer, Megan Hoffer, Keith Boniface

**Affiliations:** The George Washington University School of Medicine and Health Sciences, Department of Emergency Medicine, Washington, DC

**Keywords:** arterial ultrasound, emergency department, point-of-care ultrasound

## Abstract

**Introduction:**

The use of point-of care ultrasound (POCUS) in the evaluation of vascular emergencies including abdominal aortic aneurysm and deep vein thrombosis is well established. However, no current guidelines exist to outline the use of POCUS in the management of acute peripheral arterial pathology.

**Case Series:**

Here, we present a case series that illustrates the utility of POCUS in the assessment of both traumatic and nontraumatic peripheral arterial disease. Direct visualization of the vasculature via B-mode, color Doppler, and pulsed-wave Doppler assisted in the diagnosis of the following: 1) an acute, post-catheterization thrombus of the proximal radial artery; 2) a complete, traumatic radial artery transection; 3) a forearm hematoma with active arterial extravasation; 4) a traumatic arteriovenous fistula; 5) an acute thrombosis of an artery bypass graft; and 6) an infected pseudoaneurysm.

**Conclusion:**

The incorporation of POCUS into patient care allowed for rapid identification of significant peripheral arterial pathology and led to changes in clinical management, expedited patient care, and circumvented potentially harmful complications.

## INTRODUCTION

The use of point-of-care ultrasound (POCUS) has expanded greatly since the introduction of the focused assessment with sonography for trauma exam.^1^ The American College of Emergency Physicians’ Emergency Ultrasound Guidelines include indications for POCUS such as trauma, pregnancy, cardiac and hemodynamic assessment, soft tissue infection, musculoskeletal injury, hepatobiliary and gastrointestinal pathology, ocular assessment, genitourinary complaints, and procedural guidance.^2^ Other well-established indications for POCUS include vascular emergencies such as abdominal aortic aneurysm (AAA) and deep vein thrombosis (DVT). With sensitivity and specificity values approaching 100% for the detection of AAA and similar values for DVTs (95% and 96%, respectively), POCUS has been established as a highly effective imaging modality in the rapid assessment of vascular pathology.^3^–^8^

Despite the efficacy of POCUS in these vascular emergencies, currently no published guidelines exist that outline the use of POCUS in the assessment and management of peripheral arterial pathology. Current practice for the rapid assessment of arterial pathology, both traumatic and non-traumatic, includes obtaining a history, physical examination, ankle-brachial index, and handheld (continuous-wave) Doppler evaluation. However, given the documented utility of POCUS in the real-time evaluation of other vascular pathology, hemodynamic assessment, and procedural guidance (eg, vascular access), it would likely prove to be extremely beneficial in the diagnosis of peripheral arterial emergencies as well.

In this case series, we aim to demonstrate the value of POCUS in the early detection of acute peripheral arterial disease within the emergency department (ED). Through the clinical cases outlined below, we illustrate instances in which the use of POCUS revealed significant arterial pathology that ultimately changed clinical management, facilitated specialist consultation, and expedited definitive care.

## CASE SERIES

### Case 1

A 56-year-old female with a past medical history of diabetes mellitus, hypertension, and hyperlipidemia presented to the ED with three days of right wrist pain that began on her way home following a cardiac catheterization via her right radial artery. The pain was described as severe, constant, radiating to her right forearm and shoulder, and 10/10 in severity. It was painful to move and tender to palpation but was improved by rest and acetaminophen. The patient denied any redness, swelling, tingling, numbness, or paresthesia.

On physical examination, she had a blood pressure (BP) of 144/77 millimeters of mercury (mm Hg), heart rate (HR) of 87 beats per minute (bpm), and she was afebrile. She was in no acute distress. Her right hand was warm, appeared symmetric to her left hand, and had no edema. She had tenderness to palpation over the right lateral forearm. Her right radial artery pulse was not palpable distal to the puncture site. All other pulses including the lower limb pulses were palpable bilaterally. Neurologic examination demonstrated mild subjective sensory loss over the right radial and median nerves distributions with no motor deficits.

Basic laboratory investigation and electrocardiography were normal. A POCUS of the right upper extremity arterial vasculature was performed given concern for vascular compromise. The distal portions of the right brachial artery, including its bifurcation into the radial and ulnar arteries were patent with normal flow, as was the entire right ulnar artery. However, we found clear sonographic evidence of an occlusive thrombus in the distal right radial artery, just proximal to the access site (Image 1a). There was no apparent flow within the proximal and mid portions of the radial artery due to outflow occlusion (Image 1b). Minimal retrograde flow was noted in the right ulnar artery distal to the access site—likely supplied by the palmar arch.

The interventional cardiology and interventional radiology services were consulted. Supported by the sonographic evidence of an acute arterial thrombus, along with evidence of perfusion of the hand via collateral flow, the involved services made a collaborative recommendation to begin anticoagulation with supportive therapy. The patient was started on enoxaparin and oral analgesia as conservative management for three months, with follow-up scheduled one week from the start of her therapy.

Diagnosis: Acute post-procedural thrombus of the proximal radial artery.

**CPC-EM Capsule**

What do we already know about this clinical entity?

*Point-of-care ultrasound (POCUS) is a useful diagnostic tool in the identification of acute arterial pathology and vascular emergencies in the Emergency Department*.

What makes this presentation of disease reportable?

*Despite the efficacy of POCUS in vascular emergencies such as abdominal aortic aneurysm, currently no published guidelines exist which outline the use of POCUS in the assessment and management of peripheral arterial pathology*.

What is the major learning point?

*This case series illustrates the utility of POCUS for the diagnosis of several peripheral arterial emergencies, including arteriovenous fistula, pseudoaneurysm, and arterial thrombus*.

How might this improve emergency medicine practice?

*Given the documented utility of POCUS in the real-time evaluation of other vascular pathology, hemodynamic assessment, and procedural guidance (eg, vascular access), it would likely prove to be extremely beneficial in the diagnosis of peripheral arterial emergencies as well*.

### Case 2

A 45-year-old right hand dominant male with no significant past medical history presented to the ED after sustaining a power saw injury to the volar surface of his right forearm. While he was cutting overhead, the saw unexpectedly recoiled, striking his face and arm. Prior to transport, bleeding was controlled by emergency medical services (EMS) personnel using direct pressure. There were no hard or soft signs of vascular injury reported by EMS personnel.

Upon arrival to the ED, the patient had a BP of 130/87 mm Hg, HR of 76 bpm, temperature of 98° Fahrenheit, and an oxygen saturation (SpO2) of 96% on room air as measured on the right arm. Initial assessment revealed a four-centimeter (cm) gaping, oblique laceration through the volar aspect of his right forearm with no active hemorrhage. The patient was able to move all digits, but there was weakness of flexion of the second through fourth digits that localized to the flexor digitorum superficialis for the second through fourth digits; in addition, the flexor carpi radialis was compromised. The remainder of tendons were intact. Vascular assessment revealed a 2+ palpable pulse in the ulnar artery with no palpable radial pulse. The vascular surgery service was consulted and, using a handheld Doppler, demonstrated pulsatile flow in the radial artery.

Given the evidence of pulsatile flow with conventional handheld Doppler evaluation, the initial treatment plan was for operative repair of the tendon injuries by orthopedic surgery. However, POCUS revealed anterograde flow within the ulnar artery (Image 2a) but retrograde flow through the distal radial artery, indicating filling via the palmar arches due to radial artery interruption (Image 2b).

With this new sonographic finding, the patient was taken to the operating room (OR) for a joint vascular and orthopedic surgical operation involving saphenous vein grafting of the right radial artery in addition to the originally planned tendon repair.

Diagnosis: Complete arterial transection of the radial artery.

### Case 3

A 60-year-old female with history of hypertension, hyperparathyroidism, generalized anxiety disorder, and left-sided total knee replacement (complicated by left lower extremity DVT two months prior, treated with enoxaparin and warfarin) presented to the ED with three days of right forearm pain and swelling. The pain was sudden onset and a 10/10 in severity. There was associated numbness and weakness of the right hand. Her pain was worsened with movement and relieved by placing her right hand and wrist in a flexed position.

Two days prior to her presentation, she had been evaluated at an outside hospital and discharged home with seven days of trimethoprim/sulfamethoxazole for a presumptive diagnosis of cellulitis. A right upper-extremity venous duplex ultrasound performed during that evaluation revealed no evidence of DVT.

On physical examination, the patient had a BP of 127/84 mm Hg, HR of 89 bpm, SpO2 of 100% on room air, and was afebrile. Her right hand and wrist were held in a flexed position, and pain was elicited with passive extension of her right second, third, and fourth digits. There was a noticeable area of swelling with overlying ecchymosis at the distal, volar aspect of her right forearm. The area of swelling was tense and tender to palpation.

Labs were significant for an international normalized ratio of 1.9 (reference range: 0.8–1.1) but were otherwise unremarkable. A right upper-extremity computed tomography (CT) was obtained and identified evidence of an intramuscular hematoma within the anterior compartment of the forearm. However, the study was limited by artifact, and confirmation with ultrasound was recommended by the reading radiologist. Point-of-care ultrasound revealed pulsatile flow concerning for active extravasation at the site of a layering hematoma (Images 3a and 3b). The patient subsequently underwent ultrasound by the department of radiology, which re-identified a large hematoma in the anterior forearm with evidence of internal pulsatile flow at the level of the patient’s wrist, compatible with active internal hemorrhage or the possibility of pseudoaneurysm.

Following these radiographic findings, the patient was admitted by orthopedic surgery for neurovascular assessments every two hours with a low threshold for surgical intervention due to concern for impending compartment syndrome. Upon admission, her enoxaparin and warfarin were held, and internal medicine was consulted for updated anti-coagulation recommendations. With forearm compression and elevation, the patient symptomatically improved overnight and was discharged the following morning. Anti-coagulation recommendations from the internal medicine service included cessation of warfarin and continuation of enoxaparin for one additional month to complete her three-month treatment regimen for provoked DVT.

Diagnosis: Forearm hematoma with active arterial extravasation.

### Case 4

A 65-year-old female with a history of intravenous drug use presented to the ED with complaints of left forearm swelling. The patient stated that this swelling began recently in the area where she normally injects heroin. She reported having similar lesions in the past, which had always been “drained or cut out.” On physical examination, the patient was afebrile with a BP of 144/102 mm Hg, HR of 65 bpm, respiratory rate of 18 breaths per minute, and SpO2 99% on room air. There was a four-cm area of fluctuance over the radial aspect of the distal left forearm with multiple overlying injection sites linearly aligned. The area was absent of erythema, ecchymosis, bleeding, or drainage. Distal pulses were intact, and no sensory changes were present.

**Image 4:**
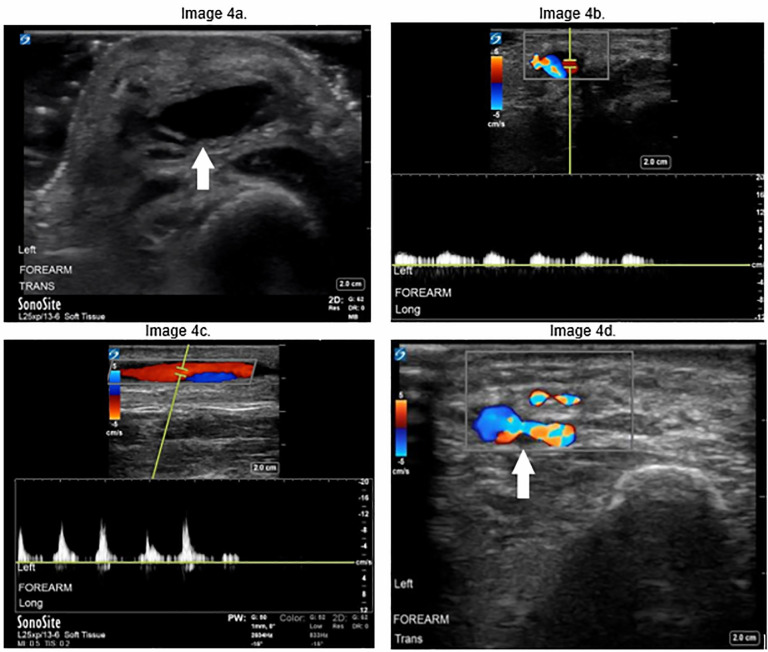
Transverse (a) view of the radial aspect of the distal forearm shows an enlarged cephalic vein (arrow) with surrounding cobblestoning. Transverse (b) and longitudinal (c) views of the cephalic vein demonstrate pulsatile flow in the vein, consistent with arteriovenous fistula; (d) Scanning distally along the cephalic vein with color Doppler demonstrates area of connection (arrow) between the radial artery (left) and vein (right).

Given the presence of fluctuance, the treatment team considered an incision and drainage, but performed POCUS of the lesion prior to intervention. The POCUS revealed a large, vascular-appearing, hypoechoic structure with surrounding cobblestoning, initially concerning for thrombophlebitis of the cephalic vein (Image 4a). Upon application of color Doppler, evidence of arterial flow pulsating in the cephalic vein and distending the vein was identified, suggesting an arteriovenous fistula, which became evident on both the transverse (Image 4b and 4d) and longitudinal (Image 4c) views.

With sonographic evidence of a traumatic arteriovenous fistula—as opposed to a suspected abscess—a potentially harmful bedside incision and drainage was avoided. Instead, the patient was scheduled to undergo CT angiography (CTA) to further characterize this vascular pathology, and plan for appropriate intervention; however, she eloped prior to CTA and vascular surgery consultation.

Diagnosis: Traumatic arteriovenous fistula of the forearm.

### Case 5

A 56-year-old male with past medical history of coronary artery disease, hypertension, type 2 diabetes, and peripheral artery disease presented to the ED with two days of right foot numbness and coolness. His symptoms began the morning following his discharge from the intensive care unit (ICU) three days prior after undergoing a right lower extremity thromboembolectomy of his femoral-posterior tibial graft. Prior to this procedure, the patient had several previous revascularizations of the same extremity. He had been discharged on warfarin, aspirin and clopidogrel, and endorsed compliance with this regimen.

On physical examination, the patient was afebrile with a BP of 122/75 mm Hg, HR of 98 bpm, respiratory rate of 16 breaths per minute, and SpO2 100% on room air. His distal right lower extremity was cool to the touch, and neither the dorsal pedis nor the posterior tibial arteries could be palpated. Capillary refill was greater than three seconds and handheld Doppler evaluation revealed no pulse. Both sensation and range of motion of the limb were intact. His left lower extremity exam was normal.

Point-of-care ultrasound was performed and revealed extensive clot burden within the bypass graft with no detectable blood flow (Images 5a and 5b).

Based on these findings the vascular surgeon recommended direct admission to the OR without any need for further imaging. The patient underwent emergent thrombectomy and alteplase thrombolytic therapy of his right lower extremity. After several days of close observation in the ICU, he was discharged with close outpatient follow-up, avoiding amputation of his limb.

Diagnosis: Acute thrombosis of femoral-posterior tibial artery bypass graft.

### Case 6

A 48-year-old male with history of intravenous drug use, deep vein thrombosis, and multiple prior soft tissue abscesses, was transferred from an outside hospital due to concern for compartment syndrome. He had undergone CT at the outside hospital, which identified an upper arm abscess. He had been having symptoms of pain, swelling, and numbness of his hand for about a week. He had not been on anticoagulation.

On physical examination at our institution, the patient was afebrile with a BP of 169/91 mm Hg, HR of 89 bpm, respiratory rate of 18 breaths per minute, and SpO2 100% on room air. Laboratory studies from the outside facility were significant for a hemoglobin of 4.7 grams per deciliter (g/dL) (reference range: 13.5–17.5 g/dL). He had a diminished radial pulse with a normal motor examination of his upper extremity. There was an upper arm wound draining seropurulent material.

Point-of-care ultrasound of the area described by CT as an abscess demonstrated turbulent swirling flow, concerning for pseudoaneurysm (Images 6a and 6b). The vascular surgery service was consulted and, given the presence of bloody drainage, took the patient emergently to the OR for incision and drainage of a distal brachial artery infected pseudoaneurysm and vein patch repair using a brachial vein patch.

## DISCUSSION

In the evaluation of arterial injury and/or occlusion, catheter-directed angiography has traditionally been the “gold standard” imaging modality of choice. However, both the costly and invasive nature of angiography as well as the potential need to call in a specialist from home has led to a shift in clinical practice favoring less invasive methods of vascular evaluation. 9,10 With improvements in CT technology, CTA is becoming the test of choice for evaluating arterial pathology. This change has become particularly evident within the ED setting due to its general availability and relatively fast turnaround times. However, CTA also carries inherent risks to the patient including radiation exposure, potential adverse reactions to intravenous contrast, and transport out of the ED.

The application of ultrasound in the assessment of peripheral arterial disease is an established technique that has been shown to provide comparable results to that of arteriography.^11^,^12^ Furthermore, its utility in rapidly identifying vascular trauma within the ED setting has also been shown.^13^–^15^ Radiology-performed ultrasound spares the patient the radiation exposure of CTA but is not available at all times in all EDs.

Point-of-care ultrasound avoids the risks of CTA while still providing vital clinical information in the evaluation of arterial injury and ischemia, and it can be performed at the patient’s bedside. Like the handheld, continuous-wave Doppler device, POCUS provides information regarding the presence and relative speed of flow. However, it is also able to provide information on the direction of flow, measure flow velocities, and display real-time visualization of the anatomy under investigation. This additional information is easy to obtain by emergency physicians with minimal training (which is not currently standard in emergency medicine residencies). By using POCUS, emergency physicians can expedite the evaluation of a patient, decreasing time to consultation and definitive treatment.

## CONCLUSION

In this series we present several cases that illustrate the utility of POCUS in the assessment of both traumatic and nontraumatic peripheral arterial disease. The incorporation of POCUS into patient care allowed for rapid identification of significant peripheral arterial pathology, led to changes in clinical management, expedited patient care, and circumvented potentially harmful complications. Thus, we argue that POCUS evaluation of peripheral arteries should replace the use of handheld (non-imaging) Doppler devices due to the additional information provided (direction of flow and B-mode image of underlying structures). Point-of-care ultrasound of peripheral arteries, and the optimization of color and pulsed-wave Doppler waveforms, should be a standard component of training for emergency physicians.

## Figures and Tables

**Image 1a:**
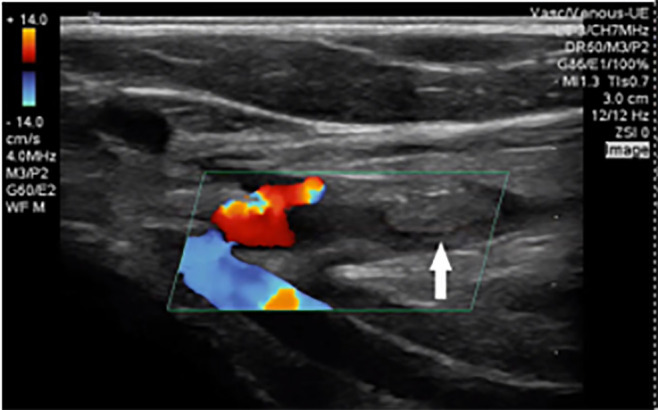
Longitudinal view of the distal brachial artery at the bifurcation into ulnar artery (deep, blue) and radial artery (superficial, red). Note the occlusive thrombus to the right of the color flow just distal to the origin of the radial artery (arrow).

**Image 1b:**
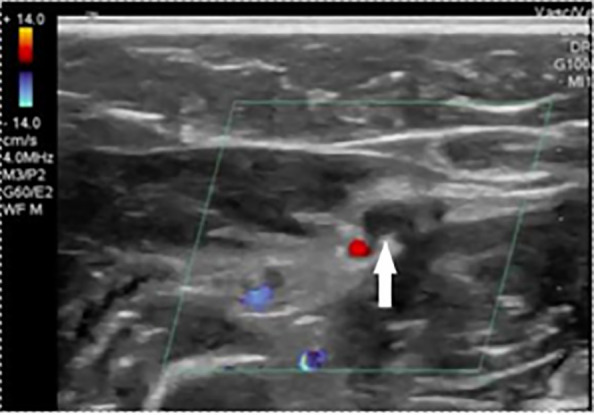
A transverse image through the distal radial artery (arrow) with absence of flow.

**Image 2a:**
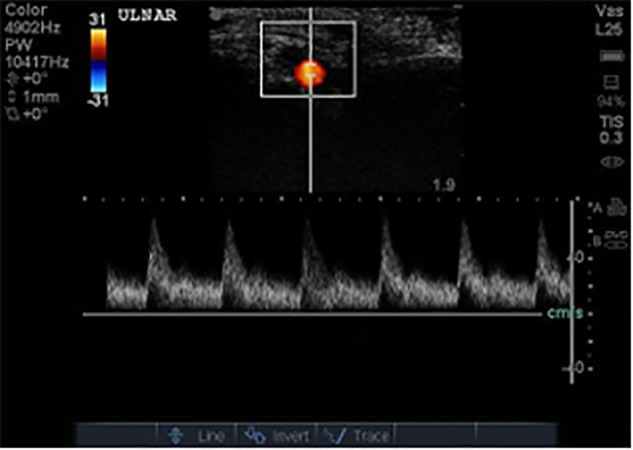
A transverse view of the ulnar artery obtained with the transducer tilted toward the patient’s proximal arm. Color Doppler and pulsed-wave Doppler reveal normal velocity anterograde flow (toward the probe face, displayed as red on color Doppler and as positive deflection above the baseline on pulsed-wave Doppler).

**Image 2b:**
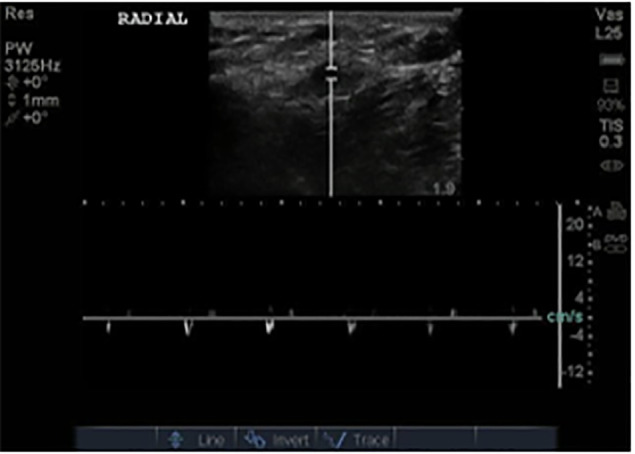
A transverse view of the radial artery distal to the injury site obtained with the transducer tilted toward the patient’s proximal arm. Color Doppler reveals minimal flow, and pulsed-wave Doppler demonstrates low velocity retrograde flow (away from the probe face, displayed as a negative deflection below the baseline), indicating flow originating from the palmar arch.

**Image 3a:**
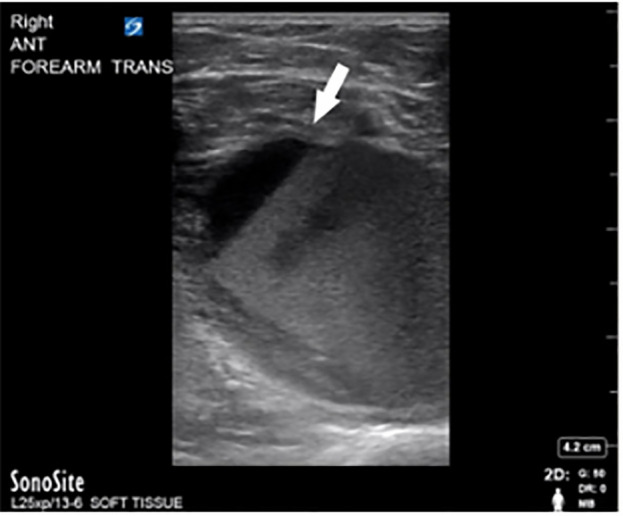
Transverse view of the mid-volar forearm demonstrates an echogenic fluid collection with fluid-debris level (arrow) consistent with a layering hematoma.

**Image 3b:**
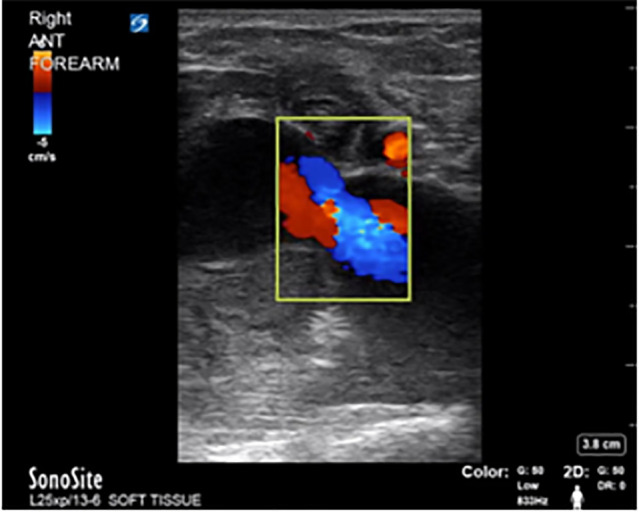
Color Doppler shows pulsatile flow into the hematoma.

**Image 5a:**
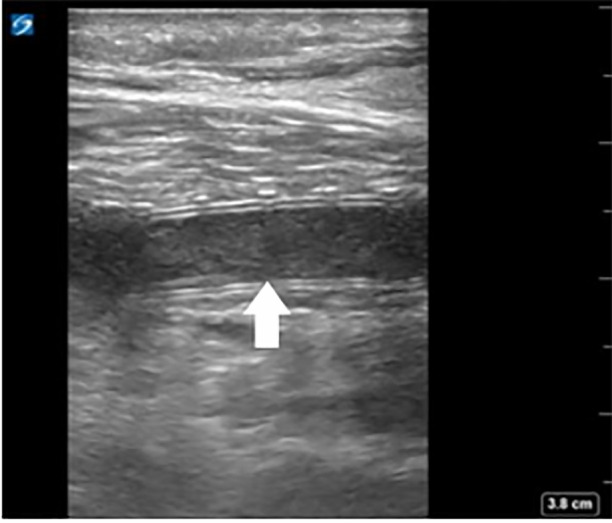
Longitudinal view of patient’s femoral-posterior tibial graft (arrow) demonstrating echogenic thrombus inside.

**Image 5b:**
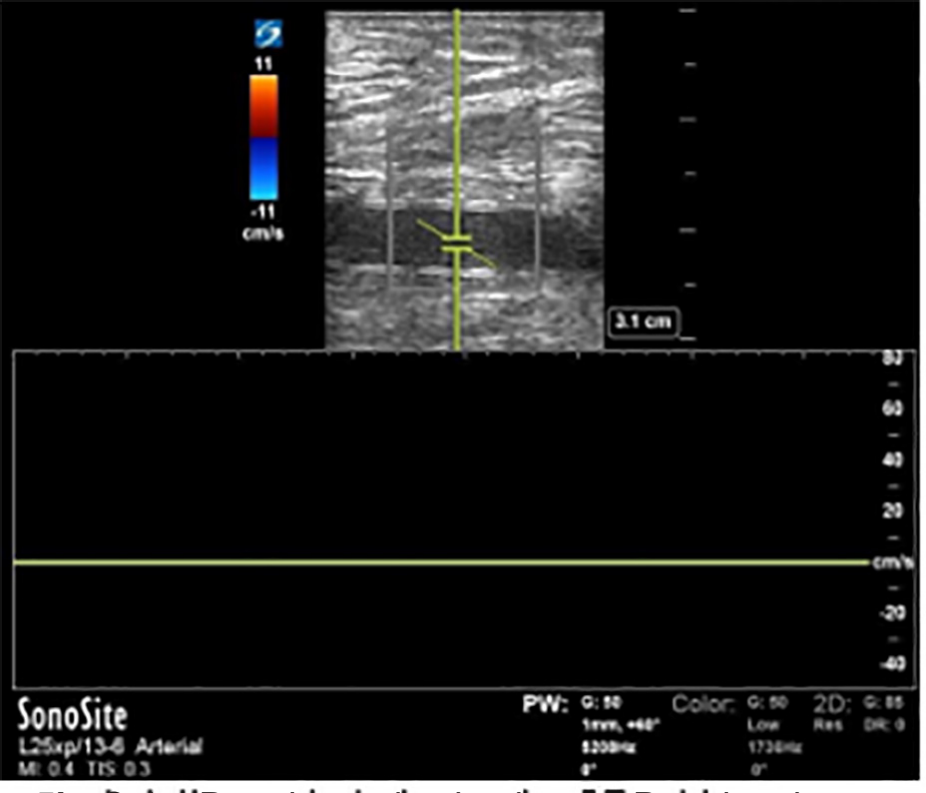
Color Doppler and pulsed-wave Doppler show no flow in this segment of thrombosed graft.

**Image 6a:**
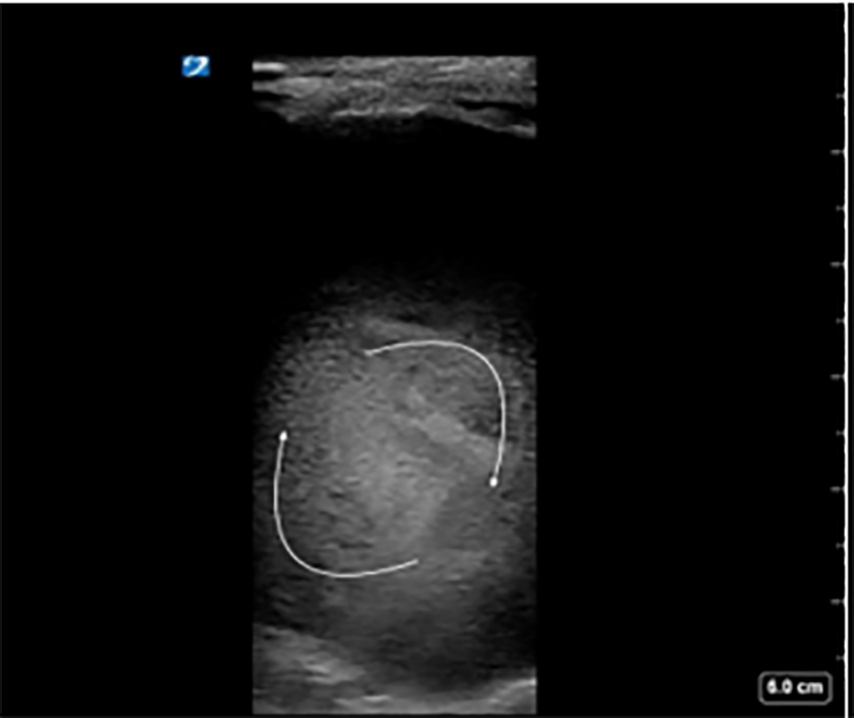
B-mode imaging of the distal medial upper arm revealed a heterogenous circular collection with demonstration of swirling of contents.

**Image 6b:**
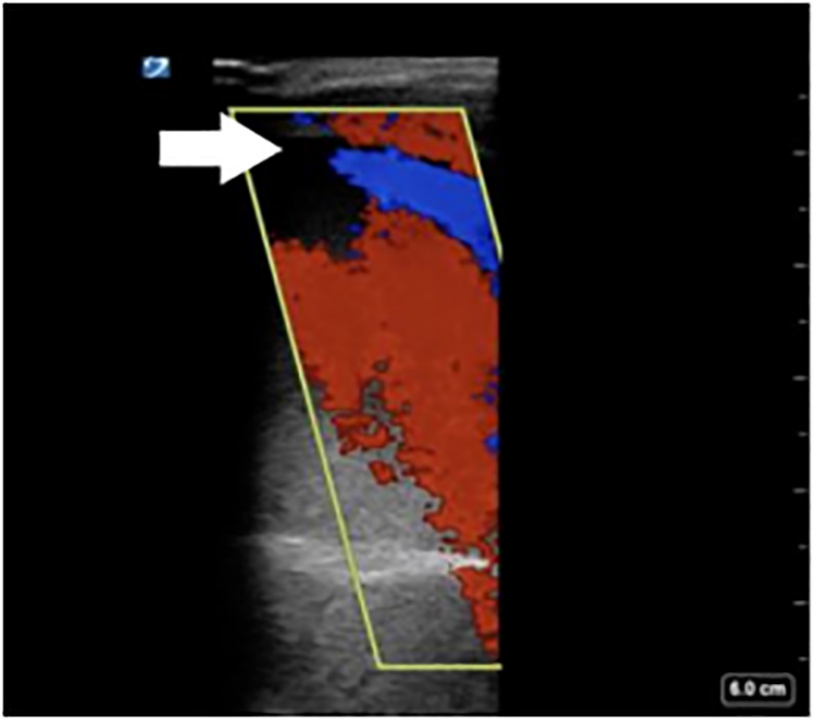
Color Doppler demonstrates swirling clockwise flow (arrow).

## References

[1] Kendall JL, Hoffenberg SR, Smith RS (2007). History of emergency and critical care ultrasound: the evolution of a new imaging paradigm. Crit Care Med.

[2] (2017). Ultrasound Guidelines: Emergency, Point-of-Care and Clinical Ultrasound Guidelines in Medicine. Ann Emerg Med.

[3] Kuhn M, Bonnin RL, Davey MJ (2000). Emergency department ultrasound scanning for abdominal aortic aneurysm: accessible, accurate, and advantageous. Ann Emerg Med.

[4] Tayal VS, Graf CD, Gibbs MA (2003). Prospective study of accuracy and outcome of emergency ultrasound for abdominal aortic aneurysm over two years. Acad Emerg Med.

[5] Rubano E, Mehta N, Caputo W (2013). Systematic review: emergency department bedside ultrasonography for diagnosing suspected abdominal aortic aneurysm. Acad Emerg Med.

[6] Burnside PR, Brown MD, Kline JA (2008). Systematic review of emergency physician-performed ultrasonography for lower-extremity deep vein thrombosis. Acad Emerg Med.

[7] Pomero F, Dentali F, Borretta V (2013). Accuracy of emergency physician-performed ultrasonography in the diagnosis of deep-vein thrombosis: a systematic review and meta-analysis. Thromb Haemost.

[8] Jang T, Docherty M, Aubin C (2004). Resident-performed compression ultrasonography for the detection of proximal deep vein thrombosis: fast and accurate. Acad Emerg Med.

[9] Miller-Thomas MM, West OC, Cohen AM (2005). Diagnosing traumatic arterial injury in the extremities with CT angiography: pearls and pitfalls. Radiographics.

[10] Allie DE, Patlola RR, Ingraldi A (2008). Peripheral vascular CTA: Emerging role of PV-CTA in the therapeutic management of PVD. Appl Radiol.

[11] Legemate DA, Teeuwen C, Hoeneveld H (1989). The potential of duplex scanning to replace aorto-iliac and femoropopliteal angiography. Eur J Vasc Surg.

[12] London NJ, Sensier Y, Hartshorne T (1996). Can lower limb ultrasonography replace arteriography?. Vasc Med.

[13] Montorfano MA, Montorfano LM, Perez Quirante F (2017). The FAST D protocol: a simple method to rule out traumatic vascular injuries of the lower extremities. Crit Ultrasound J.

[14] Montorfano MA, Pla F, Vera L (2017). Point-of-care ultrasound and Doppler ultrasound evaluation of vascular injuries in penetrating and blunt trauma. Crit Ultrasound J.

[15] Villarroel NA, Wagner W, Schoenfeld E (2019). Occult vascular transection identified by point-of-care ultrasound demonstrating evidence of retrograde flow. Clin Pract Cases Emerg Med.

